# GLUT1 and ASCT2 as Predictors for Prognosis of Hepatocellular Carcinoma

**DOI:** 10.1371/journal.pone.0168907

**Published:** 2016-12-30

**Authors:** Hong-Wei Sun, Xing-Juan Yu, Wen-Chao Wu, Jing Chen, Ming Shi, Limin Zheng, Jing Xu

**Affiliations:** 1 Sun Yat-sen University Cancer Center, State Key Laboratory of Oncology in South China, Collaborative Innovation Center for Cancer Medicine, Guangzhou, P. R. China; 2 School of Life Science, Sun Yat-sen University, Guangzhou, P. R. China; 3 Department of Hepatobiliary Oncology, Sun Yat-sen University Cancer Center, Guangzhou, P. R. China; University of North Carolina at Chapel Hill School of Medicine, UNITED STATES

## Abstract

An emerging hallmark of cancer is reprogrammed cellular metabolism, and several cancers involve increased glucose intake and glutamine addiction. Hepatocellular carcinoma (HCC) is one of the most fatal cancers, and its molecular basis needs to be delineated to identify biomarkers for its potential treatment without resection. Therefore, this study aimed to determine the metabolism status of HCC by evaluating the expression of the glucose transporter GLUT1 and glutamine transporter ASCT2. We enrolled 192 patients with surgically resected HCC in this study. Their tissue samples were subjected to immunohistochemistry to detect GLUT1 and ASCT2 expression. The prognostic value of GLUT1 and ASCT2 expression and their combined metabolic index was determined by Kaplan–Meier analysis and the Cox proportional hazards model. We found that GLUT1 and ASCT2 expression was significantly upregulated in tumor tissues as compared to adjacent non-tumor tissues and was positively associated with tumor size. Survival analysis revealed that patients with high GLUT1 or ASCT2 expression had poor overall survival (OS) and recurrence-free survival (RFS). In HCC patients, ASCT2 expression was an independent negative prognostic factor for OS (hazard ratio [HR], 1.760; 95% confidence interval [CI] = 1.124−2.755; *p* = 0.013) and the metabolic index was an independent negative prognostic factor for OS (HR = 1.672, 95% CI = 1.275−2.193, *p* < 0.001) and RFS (HR = 1.362, 95% CI = 1.066−1.740, *p* = 0.013). In conclusion, the tumor metabolism status determined by expression of GLUT1 and ASCT2 and their metabolic index is a promising prognostic predictor for HCC patients.

## Introduction

Hepatocellular carcinoma (HCC) is one of the most fatal cancers and a serious public health problem, with an increasing incidence and mortality worldwide [1]. Despite improved diagnostic and treatment strategies, surgical resection is still the most effective curative therapy for HCC [2, 3], and only a few drugs are available for the treatment of patients with unresectable HCC [4]. Therefore, it is vital to elucidate the molecular basis of HCC in order to help identify biomarkers to predict clinical outcomes of HCC patients and targets for its treatment.

To support cell growth and survival, cellular metabolism is reprogrammed to balance biosynthetic processes with energy supply to cancer cells, which is a hallmark of cancer [5]. Glucose, a fundamental source of energy, is metabolized by aerobic glycolysis in many cancers, regardless of the oxygen availability. This phenomenon, known as the Warburg effect, is accompanied by increased glucose intake [6]. Glutamine, another important nutrient and the most abundant amino acid in the serum, acts as a major source of nitrogen for nucleotide and amino acid synthesis and a source of carbon for replenishment of tricarboxylic acid cycle intermediates. Although glutamine is a nonessential amino acid in normal cells, its addiction, which is characterized by poor cancer-cell survival in the absence of glutamine, is observed in several cancers [7].

Membrane transporters, with their complicated metabolic networks, are necessary channels for nutrients influxes and may provide insight into the cellular metabolism. Glucose diffusion into the cytoplasm is facilitated by a group of membrane proteins termed glucose transporters [8]. The most typical one, glucose transporter 1 (GLUT1), is upregulated in several cancers such as colorectal cancer [9], breast cancer [10] and prostate cancer [11]. Glutamine is imported into the cytoplasm by four major transporters [12], of which the alanine-, serine-, cysteine-preferring transporter 2 (ASCT2) is essential for glutamine uptake by tumor cells [13]. ASCT2 expression was found to be upregulated in several cancers including colorectal cancer [14], breast cancer [15], and non-small cell lung cancer [16].

In this study, we aimed to delineate the metabolism status of HCC tissue by evaluating GLUT1 and ASCT2 expression and to analyze the prognostic significance of expression of these transporters in HCC patients.

## Materials and Methods

### Patients and samples

Archived, formalin-fixed, paraffin-embedded, paired tumor and non-tumor tissues were obtained from 15 patients who underwent HCC resection between 2012 and 2013 at the Sun Yat-sen University Cancer Center (Guangzhou, China). A cohort of 192 patients who underwent curative resection for HCC between 2005 and 2008 were randomly enrolled for the prognostic study. The eligibility criteria of patients’ enrollment were as follows: (a) histologically confirmed diagnosis, (b) no distant metastasis, (c) no anticancer therapies prior to surgery, (d) no serious complications or other malignant diseases, and (e) availability of resection tissues and follow-up data. The tumor stage was determined according to the 7th Edition tumor-node-metastasis classification system and the Barcelona Clinic Liver Cancer staging system. Tumor differentiation was graded according to the Edmondson grading system.

This study was approved and supervised by the Research Ethics Committee of Sun Yat-sen University Cancer Center in strict accordance with the ethical guidelines of the Declaration of Helsinki. All samples were collected after obtaining written informed consent from all patients prior to surgery. All samples were coded, and the data were stored anonymously.

### Follow-up

The follow-up procedures from our previous study were adopted [17]. Data were censored at the last follow-up for patients without recurrence or death. Overall survival (OS) was defined as the interval between the time of surgery to death or the last follow-up. Recurrence-free survival (RFS) was defined as the time from the date of surgery to recurrence, the last follow-up for patients without recurrence, or death if no recurrence was observed.

### Tissue section and microarray

Paraffin-embedded sections from the 15 patients were used to detect GLUT1 and ASCT2 expression in tumor and matched, adjacent, non-tumor tissues by immunohistochemistry (IHC). The paraffin-embedded sections were prepared as described previously [17]. In brief, formalin-fixed tissues were paraffin-embedded, cut into 4-μm sections using a microtome, and dried.

Tissue microarray (TMA) sections from the 192 patients’ specimens were used for survival analysis. The TMA was constructed as described previously [17]. Briefly, each representative area was pre-marked in the paraffin-embedded blocks on the basis of the hematoxylin and eosin (H&E) staining, excluding necrotic and hemorrhagic areas. To ensure reproducibility and homogeneity, duplicates of 1-mm (diameter) cylinders from the tumor tissues were obtained from each patient.

### IHC

IHC was performed as described in our previous study [17]. In short, the sections were sequentially deparaffinization and re-hydrated with xylene and a decreasing ethanol series. Subsequently, the slides were soaked in 0.3% H_2_O_2_ for 10 min to reduce endogenous peroxidase activity and boiled in 10 mM citrate buffer (pH 6.0) for 10 min for heat-induced epitope retrieval. The sections were then incubated with anti-GLUT1 antibody (dilution 1:500, Millipore, Cat# 07–1401) or anti-ASCT2 antibody (dilution 1:500, Novus, Cat# NBP1-89327) overnight at 4°C. Horseradish peroxidase-conjugated anti-rabbit/mouse Dako REAL^™^ EnVision^™^ detection systems (Dako, Cat# K5007) were used according to the manufacturer’s instructions. Brown color indicated positive staining. All sections were counterstained with Mayer’s hematoxylin and mounted with a non-aqueous mounting medium.

### Automated image acquisition and quantification

To assess the expression level of GLUT1/ASCT2, the Vectra-Inform image analysis system (Perkin-Elmer Applied Biosystems) was used as described in previous studies [18, 19]. In brief, a scanning protocol was designed on the basis of the TMA core number and size. Nuance multispectral image cubes (8-bit) were acquired from the TMA at a constant 200× magnification. The spectral library containing two chromogens was built with two control HCC tissue slides stained with only 1 chromogen (3,3′-diaminobenzidine or hematoxylin). The spectral library was used to unmix the signals on the multicolored images by recognizing their unique spectral curves for quantitation. Images were analyzed using InForm image analysis software (version 2.0.1; Perkin-Elmer Applied Biosystems) to identify tissue compartments (tumor and non-tumor tissue) and subcellular compartments (nucleus, cytoplasm, and membrane). The H-score was calculated by quantifying target signals in selected tissues and cellular compartments of interest following the manufacturer’s protocol.

### Statistical analysis

IBM SPSS Software (version 21; IBM Corporation) and GraphPad Prism (version 6; GraphPad Software) were used for the statistical analysis. The significance of differences between values was determined by the Wilcoxon matched-pairs signed rank test or Mann–Whitney *t*-test, as indicated. The correlation between the GLUT1/ASCT2 expression status and the clinicopathological features was analyzed by *χ*^2^ test or Fisher’s exact test, as appropriate. The Pearson correlation was used to analyze the correlation between GLUT1 and ASCT2 staining scores. The OS and RFS curves were generated by the Kaplan–Meier method and analyzed using the log-rank test. Prognostic factors were determined by univariate and multivariate analysis with the Cox proportional hazards model. Variables associated with survival by univariate analysis were adopted as covariates multivariate Cox proportional hazards analysis. A *p* value < 0.05 was considered statistically significant.

## Results

### GLUT1 and ASCT2 expression in tumor and non-tumor tissues of HCC patients

**Fig 1:**
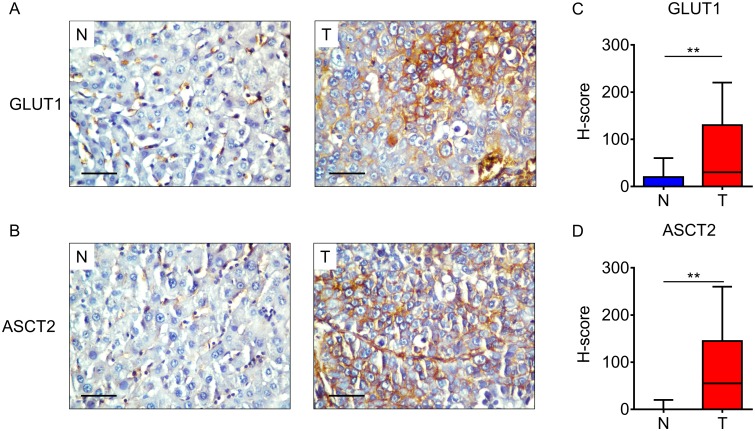
Significantly elevated GLUT1 and ASCT2 expression levels in hepatocellular carcinoma (HCC). (A–B) Immunohistochemistry (IHC) assays of GLUT1 (A) and ASCT2 (B) expression in tumor (T) and adjacent non-tumor tissues (N). The scale bar indicates 50 μm. (C-D) GLUT1 (C) and ASCT2 (D) expression levels in tumor (T) tissue are significantly higher than those in adjacent non-tumor tissue (N) (n = 15). The IHC H-scores are shown as a box-plot; ** indicates *p* < 0.01.

IHC staining of the 15 HCC specimens showed clear and distinguishable membrane staining for both GLUT1 and ASCT2 in tumor tissues, but weak staining in adjacent hepatocytes (Fig 1A and 1B). GLUT1 expression was significantly higher in tumor tissues (median score = 30.0) than in the adjacent non-tumor tissues (median score = 0.0; *p* = 0.008; Fig 1C), as quantified by the IHC H-score. A similar result was obtained for ASCT2 (median score: tumor tissues [T] = 55.0, non-tumor tissues [N] = 0.0; *p* = 0.009, Fig 1D). These data suggest that GLUT1 and ASCT2 expression was upregulated during neoplasia.

### GLUT1/ASCT2 expression and its correlation with clinicopathological characteristics

The clinicopathological features of the cohort 192 HCC patients are summarized in Table 1. The median follow-up duration was 56.2 months (range, 2−96 months). During the follow-up period, 95 patients (49.5%) died and 104 patients (54.2%) were diagnosed with tumor recurrence. The median OS and RFS for patients were 56 and 20 months, respectively. Patients were further divided into groups according to the level of GLUT1 and ASCT2 expression. The cut-off value of GLUT1 and ASCT2 expression was 31.75 and 78.75, respectively, as determined by the receiver operating characteristic (ROC) curve. GLUT1 and ASCT2 were highly expressed in 54.7% (105/192) and 35.9% (69/192) of all cases, respectively.

**Table 1:** Clinical characteristics of patients with hepatocellular carcinoma.

Variable	No. of patients	%
No. of patients	192	100.0
Age; median (range), yr	48 (13–76)	
Gender		
Female	25	13.0
Male	167	87.0
HBsAg		
Negative	20	10.4
Positive	172	89.6
AFP; median (range), ng/mL	205.7 (0.0–121000.0)	
ALT; median (range), U/L	39.0 (4.0–377.0)	
Tumor size; median (range), cm	6.0 (1.3–20.0)	
Liver cirrhosis		
No	54	28.1
Yes	138	71.9
Child–Pugh class		
A	179	93.2
B	12	6.3
C	1	0.5
Tumor number		
Single	140	72.9
Multiple	52	27.1
Satellite nodule		
No	172	89.6
Yes	20	10.4
Tumor capsule		
No/incomplete	125	65.1
Complete	67	34.9
Tumor differentiation		
I	11	5.7
II	110	57.3
III	68	35.4
IV	3	1.6
Vascular invasion		
No	174	90.6
Yes	18	9.4
TNM stage		
I	128	66.7
II	23	12.0
III	41	21.4
BCLC stage		
0	4	2.1
A	136	70.8
B	32	16.7
C	20	10.4

Abbreviations: HBsAg, hepatitis B surface antigen; AFP, α-fetoprotein; ALT, alanine aminotransferase; TNM, tumor-node-metastasis; BCLC, Barcelona Clinic Liver Cancer

**Fig 2:**
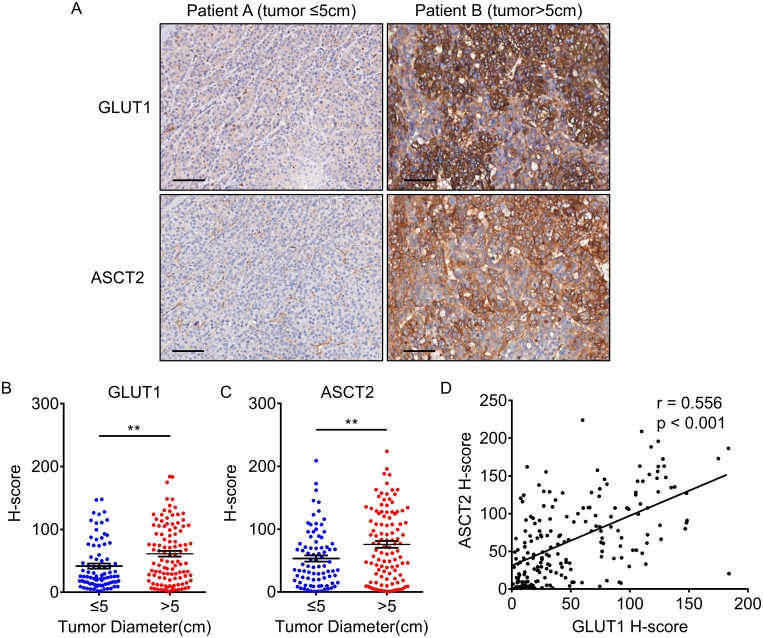
Association between expression levels of GLUT1 and ASCT2 and tumor size. (A) IHC staining for GLUT1 and ASCT2 in patients with tumor diameter less or more than 5 cm. The scale bar indicates 100 μm. (B-C) The IHC H-scores for GLUT1 (B) and ASCT2 (C) in patients with tumor diameter less or more than 5 cm. The IHC H-scores are expressed as mean ± standard error of mean (bars); ** represents *p* < 0.01. (D) GLUT1 expression level is positively correlated with ASCT2 expression (n = 192).

We also analyzed the correlation between GLUT1/ASCT2 status and clinicopathological features. Expression of both GLUT1 and ASCT2 was associated with tumor size (*p* < 0.01, Table 2). Patients with a large tumor tended to have higher expression of GLUT1 or ASCT2 (*p* < 0.01, Fig 2A−2C). In addition, ASCT2 expression was associated with poor differentiation (*p* = 0.020, Table 2). Furthermore, there was a positive correlation between GLUT1 and ASCT2 expression (r = 0.556, *p* < 0.001, Fig 2D).

**Table 2:** Correlation between GLUT1/ASCT2 expression and clinicopathological parameters.

Characteristics	No.	GLUT1		*p* Value	ASCT2		*p* Value
		Low	High		Low	High	
Age (years)							
≤48	96	44	52	1.00	60	36	0.76
>48	96	43	53		63	33	
Gender							
Female	25	14	11	0.29	19	6	0.26
Male	167	73	94		104	63	
HBsAg							
Negative	20	8	12	0.64	14	6	0.63
Positive	172	79	93		109	63	
AFP (ng/mL)							
≤20	63	32	31	0.35	46	17	0.08
>20	129	55	74		77	52	
ALT (U/L)							
≤40	100	47	53	0.67	62	38	0.55
>40	92	40	52		61	31	
Tumor size (cm)							
≤5	84	49	35	**0.001**	64	20	**0.002**
>5	108	38	70		59	49	
Liver cirrhosis							
No	54	26	28	0.63	35	19	1.00
Yes	138	61	77		88	50	
Child–Pugh class							
A	179	81	98	1.00	118	61	0.07
B-C	13	6	7		5	8	
Tumor number							
Single	140	60	78	0.74	91	49	0.74
Multiple	52	25	27		32	20	
Satellite nodule							
No	172	78	94	1.00	113	59	0.22
Yes	20	9	11		10	10	
Tumor capsule							
No/incomplete	125	56	69	0.88	77	48	0.35
Complete	67	31	36		46	21	
Tumor differentiation							
I-II	121	56	65	0.77	85	36	**0.03**
III-IV	71	31	40		38	33	
Vascular invasion							
No	174	79	95	1.00	115	59	0.08
Yes	18	8	10		8	10	
TNM stage							
I	128	58	70	1.00	86	42	0.21
II-III	64	29	35		37	27	
BCLC stage							
0-A	140	63	77	1.00	95	45	0.09
B-C	52	24	28		28	24	

*p* values were analyzed by χ2 test or Fisher’s exact test, as appropriate

Abbreviations: HBsAg, hepatitis B surface antigen; AFP, α-fetoprotein; ALT, alanine aminotransferase; TNM, tumor-node-metastasis; BCLC, Barcelona Clinic Liver Cancer; GLUT1, glucose transporter 1; ASCT2, alanine, serine, cysteine-preferring transporter 2

### Prognostic value of GLUT1 and ASCT2 expression in HCC

Univariate analysis of GLUT1/ASCT2 status and conventional clinicopathologic parameters for prognosis showed that high expression of GLUT1, high expression of ASCT2, high α-fetoprotein level, high alanine aminotransferase level, large tumor size, multiple tumor number, satellite nodule, incomplete tumor capsule, poor tumor differentiation, and vascular invasion were unfavorable predictors for OS of HCC patients (Table 3). Moreover, high expression of GLUT1, high expression of ASCT2, young age, large tumor size, multiple tumor number, poor tumor differentiation, and vascular invasion were significantly associated with shorter RFS in HCC patients (Table 3).

**Table 3:** Univariate and multivariate analysis of factors associated with survival and recurrence.

Variables	OS				RFS			
	Univariate *p* value	Multivariate			Univariate *p* value	Multivariate		
		*p* value	HR	95% CI		*p* value	HR	95% CI
Age (years)	N.S.				0.008	0.010	0.591	0.397–0.880
Gender	N.S.				N.S.			
HBsAg	N.S.				N.S.			
AFP (ng/mL)	<0.001	0.022	1.830	1.090–3.073	N.S.			
ALT (U/L)	0.006	0.002	1.946	1.280–2.959	N.S.			
Tumor size (cm)	<0.001	N.S.			0.003	N.S.		
Liver cirrhosis	N.S.				N.S.			
Tumor number	<0.001	0.011	1.881	1.159–3.057	<0.001	0.009	1.787	1.154–2768
Satellite nodule	0.022	N.S.			N.S.			
Tumor capsule	0.005	0.029	0.586	0.363–0.946	N.S.			
Tumor differentiation	0.014	N.S.			0.011	N.S.		
Vascular invasion	< 0.001	< 0.001	7.454	3.880–14.319	<0.001	<0.001	4.198	2.131–8.268
GLUT1	0.004	N.S.			0.042	N.S.		
ASCT2	< 0.001	0.013	1.760	1.124–2.755	0.026	N.S.		

Univariate analysis, Cox proportional hazards regression model.

Multivariate analysis, Cox proportional hazards regression model. Variables were adopted by univariate analysis.

Abbreviations: OS, overall survival; RFS, recurrence-free survival; HR, hazard ratio; CI, confidence interval; N.S., not significant; HBsAg, hepatitis B surface antigen; AFP, α-fetoprotein; ALT, alanine aminotransferase; GLUT1, glucose transporter 1; ASCT2, alanine, serine, cysteine-preferring transporter 2

**Fig 3:**
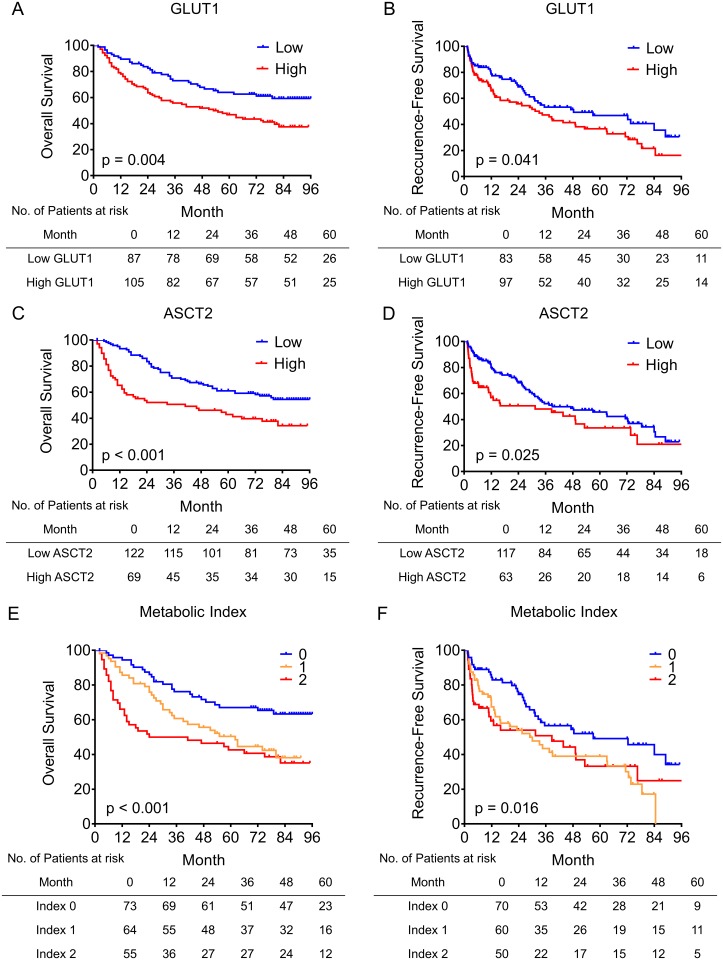
Prognostic significance of GLUT1 expression, ASCT2 expression, and the metabolic index. (A–B) HCC patients with higher GLUT1 expression have shorter OS (A) and RFS (B). (C–D) Higher expression of ASCT2 predicts poor OS (C) and RFS (D) in HCC patients. (E–F) HCC patients with higher metabolic index have poor OS (E) and RFS (F) (n = 192).

The Kaplan–Meier survival curves showed that patients with high GLUT1 expression had lower OS (*p* = 0.004, Fig 3A) and RFS rate (*p* = 0.041, Fig 3B). The 5-year OS and RFS rates of the high GLUT1 expression group were 33.6% and 17.2%, respectively, which were significantly lower than those of the low GLUT1 expression group (56.3% and 29.2%, respectively). At the same time, high ASCT2 expression indicated poorer OS (*p* < 0.001, Fig 3C) and RFS (*p* = 0.025, Fig 3D). The 5-year OS rate of the high and low ASCT2 expression group were 31.5% and 50.6%, respectively. These findings suggest that both GLUT1 and ASCT2 are important indicators of prognosis in HCC patients.

Multivariate Cox model for assessing whether GLUT1 and ASCT2 expression could be independent predictors for OS and RFS in HCC patients revealed that the ASCT2 expression was an independent negative prognostic factor for OS (HR = 1.760, 95% CI = 1.124–2.755, *p* = 0.013, Table 3) in HCC patients.

### Prognostic significance of the metabolic index

Reprogrammed cellular metabolism is a hallmark of cancer [5], in which glucose and glutamine metabolism plays a dominant role [6, 7]. GLUT1 and ASCT2 are the most important transporters for glucose and glutamine influxes and may provide insight into the cellular metabolism in cancer [8, 12, 13]. Therefore, we united GLUT1 and ASCT2 expression status into the metabolic index to evaluate the combined influence of GLUT1 and ASCT2 in HCC. Briefly, low and high expressions of GLUT1 or ASCT were assigned scores of 0 and 1, respectively. Thereafter, the GLUT1 and ASCT2 indices were added to obtain the metabolic index, which ranged from 0 to 2. Thus, patients were classified into three groups: 0, low GLUT1 and low ASCT2 (n = 73); 1, low GLUT1 and high ASCT2, or high GLUT1 and low ASCT2 (n = 64); 2, high GLUT1 and high ASCT2 (n = 55). Kaplan–Meier survival analysis revealed that patients with a high metabolism index had poor OS (*p* < 0.001, Fig 3E) and RFS (*p* = 0.016, Fig 3F). Furthermore, multivariate analysis showed that the metabolic index was an independent negative prognostic factor for both OS (hazard ratio [HR] = 1.672, 95% confidence interval [CI] = 1.275−2.193, *p* < 0.001) and RFS (HR = 1.362, 95% CI = 1.066–1.740, *p* = 0.013) in HCC patients (Table 4). Overall, the metabolic index was a powerful prognostic factor for OS and RFS in HCC patients.

**Table 4:** Univariate and multivariate analysis of metabolic index associated with survival and recurrence.

Variables	OS				RFS			
	Univariate *p* value	Multivariate			Univariate *p* value	Multivariate		
		*p* value	HR	95% CI		*p* value	HR	95% CI
Age (years)	N.S.				0.008	0.010	0.592	0.398–0.881
Gender	N.S.				N.S.			
HBsAg	N.S.				N.S.			
AFP (ng/mL)	0.001	0.020	1.842	1.100–3.086	N.S.			
ALT (U/L)	0.006	0.002	1.937	1.275–2.943	N.S.			
Tumor size (cm)	0.001	N.S.			0.003	N.S.		
Liver cirrhosis	N.S.				N.S.			
Tumor number	< 0.001	0.011	1.882	1.158–3.059	< 0.001	0.009	1.784	1.153–2.760
Satellite nodule	0.022	N.S.			N.S.			
Tumor capsule	0.005	0.029	0.586	0.363–0.947	N.S.			
Tumor differentiation	0.014	N.S.			0.011	N.S.		
Vascular invasion	< 0.001	< 0.001	7.496	3.908–14.376	< 0.001	< 0.001	4.210	2.142–8.280
Metabolic index	< 0.001	< 0.001	1.672	1.275–2.193	0.011	0.013	1.362	1.066–1.740

Univariate analysis, Cox proportional hazards regression model

Multivariate analysis, Cox proportional hazards regression model. Variables were adopted by univariate analysis.

Abbreviations: OS, overall survival; RFS, recurrence-free survival; HR, hazard ratio; CI, confidence interval; N.S., not significant; HBsAg, hepatitis B surface antigen; AFP, α-fetoprotein; ALT, alanine aminotransferase; GLUT1, glucose transporter 1; ASCT2, alanine, serine, cysteine-preferring transporter 2

## Discussion

Reprogrammed cellular metabolism is an emerging hallmark of cancer, and the altered metabolic processes are potential targets for treatment of HCC. In the present study, we detected the expression of two important transporters GLUT1 and ASCT2 to delineate their metabolic status in HCC, and evaluated their prognostic values.

Our data showed that GLUT1 and ASCT2 expression was significantly upregulated in HCC as compared to the adjacent non-tumor hepatocytes. In a previous study, GLUT1 expression was reported to be upregulated in several cancers such as colorectal cancer and cervical cancer, and its high expression was an independent factor for poor prognosis in colorectal cancer [9, 20]. A functional study suggested that GLUT1 was critical for the proliferation and migration of HCC cells [21], but its prognostic value has not yet been reported. Similarly, ASCT2 expression was found to be upregulated in several cancers, including colorectal cancer [14], breast cancer [15], non-small cell lung cancer [22], and clear-cell renal cell carcinoma [23]. Elevated ASCT2 expression promoted cell growth and survival in colorectal cancer and lung cancer, partially mediated by mammalian target of rapamycin (mTOR) signaling [14, 16]. In accordance with the vital functions of GLUT1 and ASCT2, we found that patients with higher expression of GLUT1/ASCT2 had shorter OS and RFS. Furthermore, the metabolic index, which combines the GLUT1 and ASCT2 expression status, was an independent prognostic factor for OS and RFS in HCC.

The expression levels of GLUT1 and ASCT2 were most closely related to the tumor size among the conventional clinicopathological characteristics of HCC. GLUT1 and ASCT2 are necessary for cell proliferation and tumor growth [14, 16, 21], and large tumors might promote the expression of GLUT1 and ASCT2. Large tumors are usually accompanied by a hypoxic condition, which is known to induce the expression of hypoxia-induced factor 1α (HIF1α) [24] that is associated with tumor size [25] and promotes the expression of GLUT1 in HCC [21]. Consistently, we found that the expression of GLUT1 was higher in tumors larger than 5 cm in diameter. In a previous report, lactate, a product of glycolysis, was shown to trigger the expression of ASCT2 via activation of c-Myc [26], suggesting a close relation between glucose and glutamine metabolism. In the present study, we found that ASCT2 expression was positively associated with GLUT1 expression. These data suggest a positive feedback loop between tumor growth and GLUT1/ASCT2 upregulation, which might be important for HCC progression.

Glucose and glutamine are important nutrients for not only cancer cell survival, but also immune cell function. For example, they are important for T-cell activation and differentiation [27]. When deprived of glucose, T cells display defective cell activation and functional capacity, such as impaired clonal expansion and reduced interferon (IFN)-γ, granzyme, and perforin production [28–30]. Under conditions of glutamine deprivation, naïve CD4^+^ T cells preferably differentiate into Foxp3^+^ regulatory T (T_reg_) cells, even if activated by cytokines that normally induce the generation of T helper 1 (T_H_1) cells [31]. Due to strong nutrient influx in cancer cells, GLUT1 and ASCT2 may contribute to development of a tumor microenvironment by regulating the nutrient concentration. In recent studies, several research groups independently showed that the Warburg metabolism enables tumor cells to restrict glucose availability to T cells, suppressing the anti-tumor immunity [32–34]. Thus, the nutrient competition is a new avenue of tumor immune suppression in addition to the immune checkpoints [35].

In conclusion, GLUT1 and ASCT2 expression was significantly upregulated in HCC tumor cells as compared to adjacent non-tumor hepatocytes, especially in large tumors. Patients with high GLUT1 or ASCT2 expression have poor OS and RFS. Furthermore, the metabolic index was an independent prognostic factor for both OS and RFS. Our study suggests that the tumor metabolism status determined by GLUT1 and ASCT2 expression might be a promising prognostic biomarker and therapy target in HCC patients.

## Supporting Information

S1 Supporting Information Detailed information of patients with HCC. (SAV)

## Abbreviations

AFP: α-fetoprotein
ALT: alanine aminotransferase
ASCT2: alanine, serine, cysteine-preferring transporter 2
CI: confidence interval
GLUT1: glucose transporter 1
HBsAg: hepatitis B surface antigen
HCC: hepatocellular carcinoma
HIF1α: hypoxia induced factor 1α
HR: hazard ratio
IHC: immunohistochemistry
N.S.: not significant
OS: overall survival
RFS: recurrence-free survival
TMA: Tissue microarray
